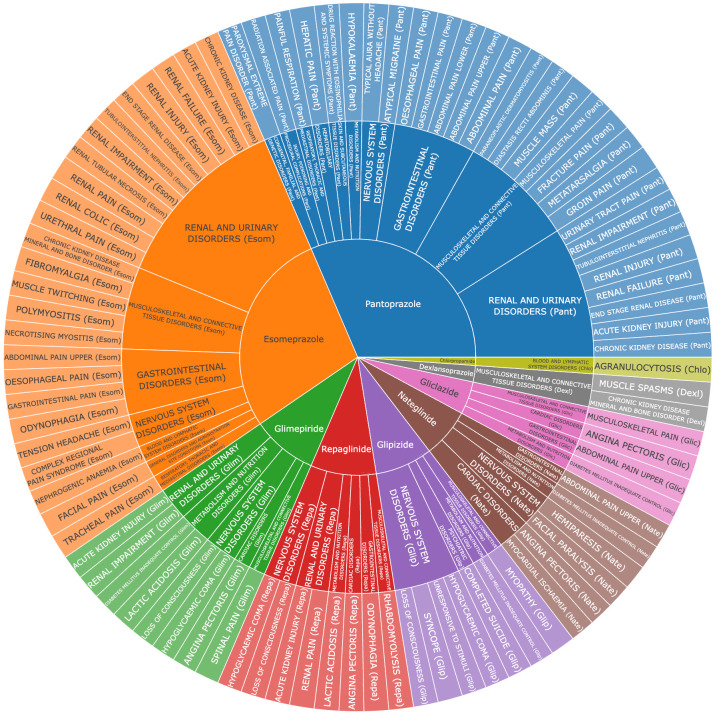# Sarcopenia and chronic pain: Identification of shared genetic determinants and therapeutic implication

**DOI:** 10.1097/MD.0000000000048819

**Published:** 2026-05-15

**Authors:** Juan Li, Jiaqi Huang, Jiang Han

**Affiliations:** a Department of Anesthesiology, West China Second University Hospital, Sichuan University, Chengdu, China; b Key Laboratory of Birth Deficits and Related Diseases of Women and Children, Sichuan University, Ministry of Education, Chengdu, China; c Key Laboratory of BioResource and Eco-Environment of Ministry of Education, College of Life Science, Sichuan University, Chengdu, China.

**Keywords:** chronic pain, genetic, sarcopenia, therapeutic

## Abstract

Sarcopenia and chronic pain are prevalent age-related conditions with substantial health impacts, yet their causal relationship remains unclear. Our aim is to study the bidirectional causal relationship among these diseases and identify potential therapeutic targets through genetic methods, as well as explore new therapeutic drugs. We performed bidirectional Mendelian randomization and Bayesian colocalization analyses using GWAS data from sarcopenia and chronic pain studies to explore their genetic relationships. Through integrating PheWAS and DrugBank analyses, we identified potential therapeutic candidates. We then evaluated these candidates using FAERS database for safety profiles and explored their pathway-level associations through drug-omics data. Our analyses revealed significant bidirectional genetic associations between sarcopenia and chronic pain, identifying 9 shared genes (*MAPKAPK3*, *MYBPC3*, *POLR2L*, *DDAH1*, *FAM177B*, *ABCC8*, *RMDN3*, *RFTN2*, and *SUOX*). Four genes (*MAPKAPK3*, *DDAH1*, *ABCC8*, and *SUOX*) were identified as druggable targets, with 18 compounds (including approved, investigational, and preclinical drugs) identified as potential therapeutic candidates. After FAERS screened and excluded candidate drugs that might aggravate muscle or pain symptoms, dexlansoprazole and glipizide showed relatively favorable safety profiles among compounds targeting these genes. Subsequent drug-omics analysis identified pathway enrichments consistent with muscle and pain-related processes, though clinical efficacy remains unestablished. This study provides genetic evidence for a causal bidirectional relationship between sarcopenia and chronic pain, identifying potential therapeutic targets. However, findings are based on computational analyses of summary-level data without experimental validation. The identified drug candidates warrant further rigorous experimental and clinical investigation for repurposing strategies in managing these conditions.

**Key Points**

- This study systematically established bidirectional causal relationships between sarcopenia and chronic pain in aging populations, filling critical gaps left by observational studies that could not determine causal direction.
- Nine key genes involved in the common pathophysiological processes of both conditions were identified, elucidating shared biological pathways including inflammatory responses, mitochondrial dysfunction, and neuromuscular signaling.
- Four druggable key targets were discovered, and 18 potential therapeutic compounds were identified through comprehensive drug repurposing analysis, with particular validation of dexlansoprazole (targeting *DDAH1*) and glipizide (targeting *ABCC8*) through rigorous pharmacomics verification and real-world safety profiling using FAERS database screening.
- Proposed innovative treatment approaches that could simultaneously address both sarcopenia and chronic pain, potentially improving treatment efficiency and patient outcomes while establishing evidence-based clinical screening frameworks to support early detection of co-occurring conditions and enable preventive interventions in high-risk populations.

## 1. Introduction

Sarcopenia is a progressive skeletal muscle disease characterized by the deterioration of both muscle quality and quantity.^[1-3]^ This condition predominantly affects the elderly population, with a prevalence rate of up to 50%, leading to frailty, cognitive impairment, and increased mortality.^[4]^ Despite growing clinical evidence suggesting potential associations with other age-related conditions, the shared genetic architecture and common therapeutic targets of sarcopenia remain poorly understood. Therapeutic interventions for sarcopenia remain limited due to its multifactorial etiology encompassing lifestyle, pathological, and genetic components.

Chronic pain, defined as persistent pain lasting over 3 months,^[5,6]^ represents another major health challenge with prevalence ranging from 15% to 65%.^[7,8]^ Importantly, the genetic basis for chronic pain susceptibility and its potential overlap with other age-related conditions remains incompletely characterized. Chronic pain is associated with decreased physical activity, reduced muscle mass and strength – factors that may contribute to sarcopenia development, suggesting potential biological interconnections between these conditions.

While the coexistence of sarcopenia and chronic pain represents an important unmet clinical need, their causal relationship and shared genetic mechanisms remain poorly understood. Epidemiological studies have yielded mixed results regarding their association. Research has demonstrated that older individuals with chronic pain exhibit a 35% higher risk of developing sarcopenia,^[9]^ with elevated chronic pain prevalence among sarcopenia individuals. Study reported higher sarcopenia prevalence in individuals with chronic pain compared to those without (68% vs 52.4%).^[10]^ Similarly, research from Greece documented increased chronic pain prevalence in sarcopenic elderly individuals (44.3%) compared to non-sarcopenic counterparts (16.53%).^[11]^ However, contrasting findings emerge from Japanese research, which found no significant association between chronic pain and sarcopenia in older adults.^[12]^ Furthermore, another study suggested that sarcopenia correlates specifically with severe pain, while moderate or mild pain showed no significant association.^[13]^ These conflicting results underscore the uncertainty regarding the causal relationship between these conditions.

To address this knowledge gap, we employed a comprehensive analytical approach. This study hypothesizes that sarcopenia and chronic pain share common genetic architecture that underlies bidirectional causal relationships between these conditions. The specific aims were to: test whether there is a causal relationship between sarcopenia traits and chronic pain using Mendelian randomization; identify shared genes and biological pathways that contribute to both conditions; and explore whether existing drug targets acting on these genes may be suitable for therapeutic repurposing.

## 2. Materials and methods

### 2.1. Data source

#### 2.1.1. Primary discovery cohorts

For sarcopenia traits, we used the largest available genome-wide association study (GWAS) for each trait as primary discovery datasets: 1 dataset for appendicular lean mass (ALM), 1 primary dataset for hand grip strength (HGS), and 1 primary dataset for walking pace (WP).^[14-16]^ For chronic pain, primary analyses utilized 1 dataset each for multisite chronic pain (MCP)^[17,18]^ and chronic widespread pain (CWP)^[19,20]^ as main discovery cohorts (Table S1, Supplemental Digital Content). These datasets represent temporal expansions of the same consortium data rather than truly independent cohorts, with estimated sample overlap of approximately 57% to 73% between discovery and validation datasets.

#### 2.1.2. Expanded-dataset confirmation and sensitivity analyses

Additional datasets were used for expanded-dataset confirmation and sensitivity analyses: 1 additional MCP dataset,^[17,18]^ 1 additional CWP dataset,^[19,20]^ and 9 site-specific pain GWAS datasets including headache, hip pain, neck/shoulder pain, stomach and abdominal pain, back pain, knee pain, and facial pain (Table S1, Supplemental Digital Content).

#### 2.1.3. Supporting databases

Expression quantitative trait loci (eQTL) data were obtained from genotype-tissue expression (GTEx; tissue-specific)^[21]^ and eQTLgen (whole blood)^[22]^ for functional annotation. The FDA Adverse Event Reporting System (FAERS) database was used for post-market safety evaluation. Detailed cohort characteristics, inclusion criteria, and quality control procedures are provided in Table S1, Supplemental Digital Content.

### 2.2. Statistical methods

#### 2.2.1. Association analyses between sarcopenia and chronic pain

**Figure 1.**
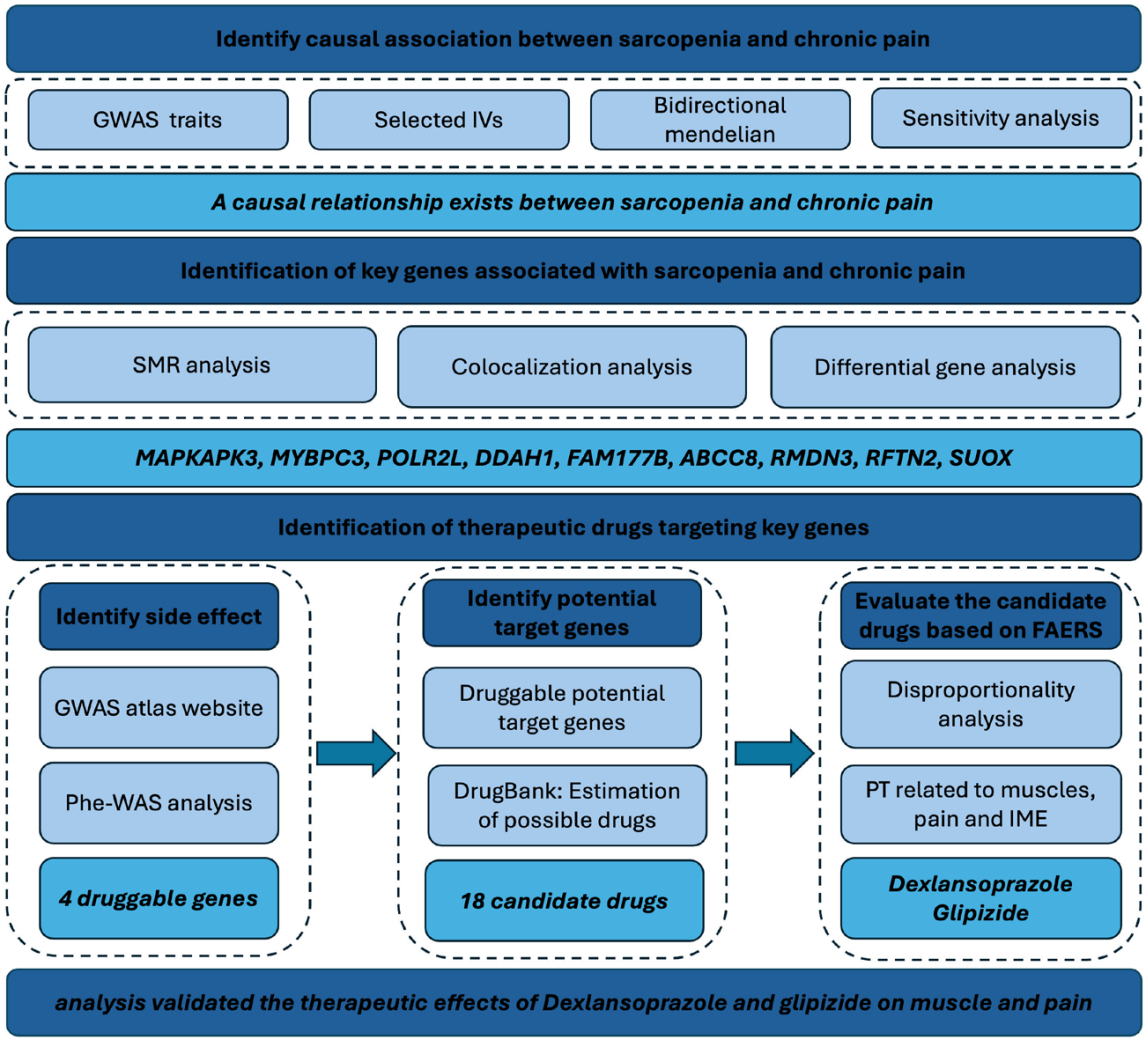
Flow diagram of the study. Definitions: Exposure: causal factor (sarcopenia/chronic pain in bidirectional analysis; cis-eQTL data in gene identification). Outcome: effect measured (chronic pain/sarcopenia in bidirectional analysis; sarcopenia/chronic pain phenotypes in gene identification). Candidate drugs: compounds potentially treating both sarcopenia and chronic pain. eQTL = expression quantitative trait loci, GWAS = genome-wide association study, IME = important medical event, IVs = instrumental variables, PheWAS = phenome-wide association study, PT = preferred terms, SMR = summary data-based Mendelian randomization.

We designed a bidirectional 2-sample MR study to investigate causal relationships between sarcopenia and chronic pain (Fig. 1). The analyses examined bidirectional relationships between 3 sarcopenia traits (hand grip strength, appendicular lean mass, walking pace) and multiple chronic pain phenotypes (multisite chronic pain, chronic widespread pain, and site-specific pain conditions).

Instrumental variables were selected using genome-wide significance criteria (*P* < 5 × 10^−6^) and clumped for independence using linkage disequilibrium threshold *R*^2^ < 0.001 within a 10,000 kb window. Palindromic single nucleotide polymorphisms (SNPs) with ambiguous allele frequencies (0.42–0.58) were excluded, and allele alignment used 1000 Genomes European reference (Table S2, Supplemental Digital Content).^[23]^

Following data harmonization, we performed bidirectional MR analysis using inverse variance weighted (IVW) as the primary method, with MR-Egger and weighted median as sensitivity analyses. Heterogeneity was assessed using Cochran *Q* statistic and horizontal pleiotropy using MR-Egger intercept test (*P* < .05 for significance). Multiple testing was controlled using Bonferroni correction (Fig. S1A, Supplemental Digital Content). All data analyses were performed using R software (version 4.4.2; R Foundation for Statistical Computing, Vienna, Austria).

To investigate associations between gene expression levels and specific traits, we employed summary data-based Mendelian randomization (SMR) using GWAS summary and gene eQTL data (Fig. S1B, Supplemental Digital Content).^[24]^ We selected the most strongly associated eQTLs using a *P*-value threshold of 5.0 × 10^−8^ and applied a 2 Mb window around the probe center for cis-eQTLs identification. The heterogeneity in the dependent instrument (HEIDI) test was performed to control for SNP heterogeneity and LD effects, with significant criteria of *P*-HEIDI > .05 and adjusted *P* < .05.

Bayesian colocalization analysis was conducted using the “coloc” R package, with an LD threshold *r*^2^ to 0.2 and a 1 Mb LD window. We defined 200 kb windows around the significant SNPs and merged adjacent windows separated by <100 kb. Loci were analyzed using COLOC with default priors, considering colocalization at posterior probability H4 > 0.75.

For transcriptome analysis, we examined differentially expressed gene (DEG) using 3 datasets: GSE226151 (60 skeletal muscle samples from healthy, pre-sarcopenic, and sarcopenic older adults), GSE1428 (vastus lateralis tissue from 10 young and 12 older males),^[25]^ and GSE221921 (96 fibromyalgia cases and 93 controls).^[26]^ GSE226151 contained 60 skeletal muscle samples from older adults (healthy, pre-sarcopenic, and sarcopenic). GSE1428 is composed of vastus lateralis muscle tissue from 10 young (19–25 years old) and 12 older (70–80 years old) males (DEG: |logFC| > 0.5). All DEG identified in GSE226151 and GSE1428 were aggregated as sarcopenia DEG. GSE221921 includes RNA sequencing results from 96 fibromyalgia cases and 93 healthy controls. Fibromyalgia (FM) is a chronic pain syndrome that is characterized by widespread chronic pain (DEG: |logFC| > 1).

#### 2.2.2. PheWAS analysis and identification of drug for potential candidate genes

Phenome-wide association study (PheWAS) analysis was performed to validate the reliability of candidate genes. The PheWAS online tool enables researchers to conduct PheWAS analysis using GWAS data from the GWAS Atlas.^[27]^ We also searched for drug and target information in the DrugBank database to explore currently existing drugs targeting our potential candidate genes.^[28]^

#### 2.2.3. Real-world pharmacovigilance analysis on adverse events of therapeutic candidate

Real-world pharmacovigilance analysis was conducted to evaluate the safety profile of candidate drugs identified through our MR analyses, providing crucial evidence for clinical translation by identifying potential muscle- and pain-related adverse events that could contraindicate their therapeutic use.

##### 2.2.3.1. AE classification and data collection

In the FAERS database, adverse events are classified using Preferred Terms (PTs) from the Medical Dictionary for Regulatory Activities. We systematically examined target drugs under their various nomenclatures (Table 1).

**Table 1:** Existing drugs searched on DrugBank database.

Gene name	Drug generic name	DrugBank accession number	Molecule type	State	Actions	Indication
MAPKAPK3	–	DB07728	Small molecule	Experimental		
	–	DB08358	Small molecule	Experimental		
DDAH1	Citrulline	DB00155	Amino acid	Investigational, nutraceutical	Inhibitor	Nutrition supplementary and treating dietary shortage or imbalance
	Pantoprazole	DB00213	Small molecule	Approved	Inhibitor	Gastroesophageal reflux disease; erosive esophagitis; Zollinger–Ellison Syndrome
	Esomeprazole	DB00736	Small molecule	Approved, investigational	Inhibitor	Acid-reflux disorders
	Dexlansoprazole	DB05351	Small molecule	Approved, investigational	Inhibitor	Erosive esophagitis; symptomatic nonerosive gastroesophageal reflux disease
ABCC8	ATP	DB00171	Small molecule	Investigational, nutraceutical	Substrate	Nutritional supplementation; treating dietary shortage or imbalance
	Gliclazide	DB01120	Small molecule	Approved	Binder	Type 2 diabetes
	Nateglinide	DB00731	Small molecule	Approved, investigational	Inhibitor	Type 2 diabetes
	Repaglinide	DB00912	Small molecule	Approved, investigational	Inhibitor	Glycemic control in adults with type 2 diabetes mellitus
	Mitiglinide	DB01252	Small molecule	Investigational	Inhibitor	Type 2 diabetes
	Glipizide	DB01067	Small molecule	Approved, investigational	Inhibitor	Glycemic control in adults with type 2 diabetes mellitus
	Tolbutamide	DB01124	Small molecule	Approved, investigational	Inhibitor	Type 2 diabetes
	Chlorpropamide	DB00672	Small molecule	Approved, investigational	Inhibitor	Type 2 diabetes
	Gliquidone	DB01251	Small molecule	Approved, investigational	Inhibitor	Type 2 diabetes
	Glimepiride	DB00222	Small molecule	Approved	Inducer	Type 2 diabetes
	Glymidine	DB01382	Small molecule	Approved, investigational	Inducer	Type 2 diabetes
SUOX	–	DB03983	Small molecule	Experimental		

##### 2.2.3.2. Signal detection analysis

We employed 4 established signal detection methods to identify disproportionate reporting patterns: reporting odds ratio (ROR),^[29]^ proportional reporting ratio (PRR),^[30]^ Bayesian confidence propagation neural network (BCPNN),^[31]^ and empirical Bayesian geometric mean (EBGM).^[32]^ Each method uses different statistical approaches to detect signals, with positive signals indicating higher-than-expected reporting rates for specific drug-AE combinations. Detailed thresholds and computational formulas are provided in Table S3, Supplemental Digital Content.

##### 2.2.3.3. AE classification

We specifically focused on muscle-related adverse events (including myalgia, muscle weakness, muscle atrophy) and pain-related adverse events (including various pain conditions) to directly assess whether candidate drugs might exacerbate the clinical conditions under study. Additionally, we classified serious adverse events using the EudraVigilance Expert Working Group’s important medical event list (version 26.1) to identify potentially life-threatening reactions.

#### 2.2.4. The omics data of dexlansoprazole and glipizide before and after medication suggested potential associations with muscle and pain-related pathways

We analyzed the GSE216991 dataset, which represents a high-throughput screen of 1902 bioactive compounds from the Selleckchem library in human iPSC-derived neurons. This study was originally designed to identify small molecule inducers of miR-132 for Alzheimer disease research. Among the screened compounds, dexlansoprazole and glipizide were included as single samples (n = 1 each), compared to DMSO negative controls (n = 8) and forskolin positive controls (n = 4). This represents an exploratory single-sample analysis comparing treated samples with untreated controls, rather than a paired pre- and posttreatment design. Differentially expressed miRNAs were identified based on expression level thresholds rather than statistical testing, given the absence of biological replicates for the candidate compounds. The treatment timing information is not explicitly provided in the source dataset. This dataset provides a resource for examining molecular changes following drug treatment that can be linked to muscle and pain-related biological pathways. We focused on differentially expressed microRNAs (miRNAs) between drug-treated samples and controls, as these regulatory molecules can reflect molecular responses at the cellular level.^[33]^ Target genes regulated by differentially expressed miRNAs were identified through miRTarBase database queries, followed by Gene Ontology (GO) and kyoto encyclopedia of genes and genomes pathway enrichment analyses to determine whether molecular changes are consistent with muscle and pain-related biological pathways, though this does not establish therapeutic efficacy.

## 3. Results

### 3.1. The causal relationship of sarcopenia on chronic pain

**Figure 2.**
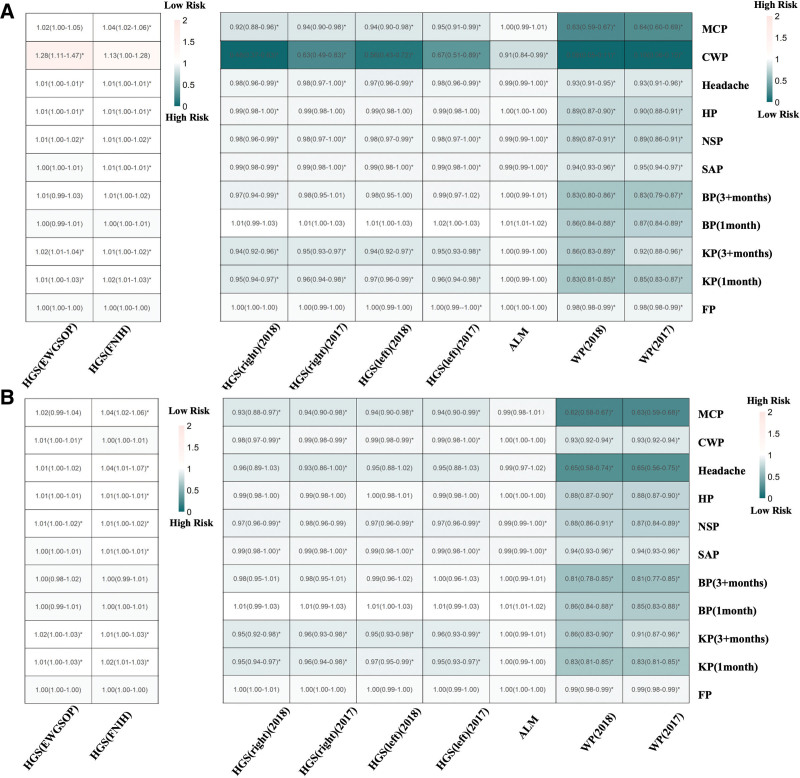
Heatmaps of bidirectional Mendelian randomization when sarcopenia served as an exposure factor. OR values > 1.0 indicate increased risk, while OR values < 1.0 indicate protective effects. For continuous variables with increasing values (e.g., muscle strength, muscle mass), this interpretation applies directly; for continuous variables with decreasing values (e.g., sarcopenia indicators), the interpretation should be reversed. The most pronounced (*P* < .05) results are marked using *. (A) Bidirectional Mendelian randomization results with sarcopenia as an exposure factor. (B) Results of bidirectional Mendelian randomization for replication. ALM = appendicular lean mass, BP = back pain, CWP = chronic widespread pain, EWGSOP = European working group on sarcopenia in older people, FNIH = Foundation for the National Institutes of Health, FP = facial pain, HGS = hand grip strength, HP = hip pain, KP = knee pain, MCP = multisite chronic pain, NSP = neck or shoulder pain, OR = odds ratio, SAP = stomach or abdominal pain, WP = walking pace.

We identified 2473, 700, and 490 SNPs associated with sarcopenia and chronic pain (*R*^2^ < 0.001, *F*-statistic > 10; Table S2, Supplemental Digital Content). Analysis of sarcopenia as the exposure factor revealed significant IVW results in 70 of 99 combinations (Fig. 2A). Hand grip strength showed consistent associations with chronic pain across different measurement approaches, though expressed in opposite directions due to variable definitions. Sarcopenia-defining low grip strength by clinical criteria (European working group on sarcopenia in older people [EWGSOP]: ORs 1.01–1.28; Foundation for the National Institutes of Health [FNIH]: ORs 1.01–1.13) increased chronic pain risk, while continuous grip strength measurements (ORs: 0.48–0.67) showed protective effects for higher values-both findings converge on the same biological relationship that muscle weakness increases pain susceptibility. Similarly, other muscle function indicators (ALM and walking pace) demonstrated protective effects (ORs: 0.08–1.00). Expanded-dataset confirmation analysis using additional chronic pain GWAS datasets with overlapping samples confirmed these associations in 59 combinations (ORs: 0.62–0.99 and 1.01–1.04; Fig. 2B). The expanded-dataset confirmation results showed consistent effect directions and overlapping confidence intervals with the discovery analysis (compare Fig. 2A, B), supporting the robustness of our findings. Heterogeneity testing (*Q P*-values > .05) supported the consistency of these findings. Pleiotropy analysis showed minimal bias, with only 3 combinations showing evidence of horizontal pleiotropy, indicating that most causal estimates are reliable (Table S4, Supplemental Digital Content).

**Figure 3.**
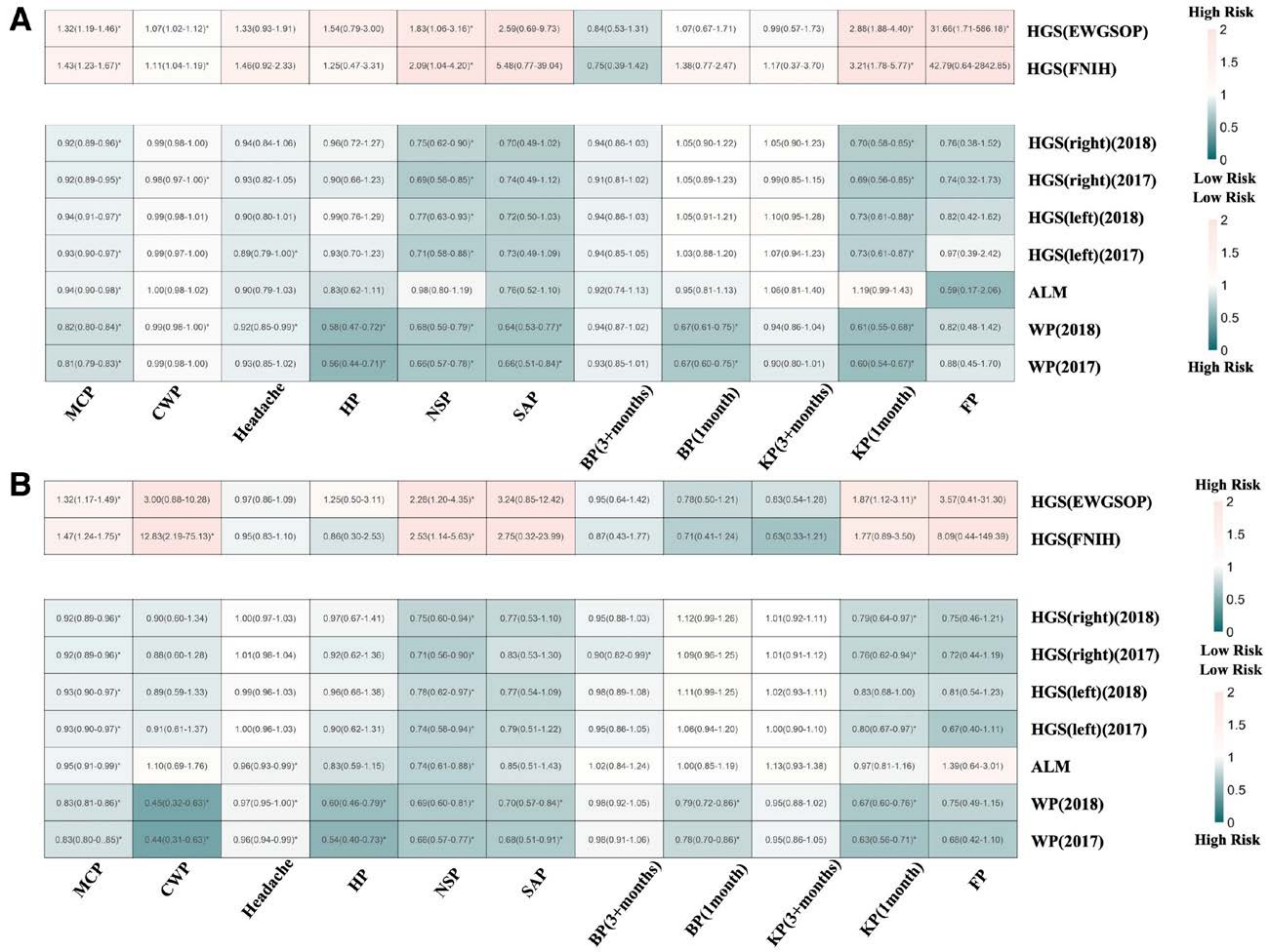
Heatmaps of bidirectional Mendelian randomization when chronic pain serves as an exposure factor. OR values > 1.0 indicate increased risk, while OR values < 1.0 indicate protective effects. For continuous variables with increasing values, this interpretation applies directly; for continuous variables with decreasing values, the interpretation should be reversed. The most pronounced (*P* < .05) results are marked using *. (A) Bidirectional Mendelian randomization results when chronic pain serves as an exposure factor. (B) Results of bidirectional Mendelian randomization for replication. ALM = appendicular lean mass, BP = back pain, CWP = chronic widespread pain, EWGSOP = European working group on sarcopenia in older people, FNIH = Foundation for the National Institutes of Health, FP = facial pain, HGS = hand grip strength, HP = hip pain, KP = knee pain, MCP = multisite chronic pain, NSP = neck or shoulder pain, OR = odds ratio, SAP = stomach or abdominal pain, WP = walking pace.

Reverse causation analysis revealed that chronic pain increases sarcopenia risk, with 38 significant associations across 99 combinations (Fig. 3A). Chronic pain demonstrated consistent detrimental effects on muscle health through complementary pathways: increasing clinical sarcopenia risk (EWGSOP: ORs 1.07–31.66; FNIH: ORs 1.43–3.21) while simultaneously impairing muscle function indicators (ORs: 0.56–0.99). These opposing OR directions reflect the same underlying biological relationship – that pain compromises muscle integrity. MCP emerged as the strongest predictor, significantly affecting all 9 sarcopenia traits (ORs: 0.81–0.94 for muscle function decline; 1.32–1.43 for sarcopenia risk increase), suggesting that widespread pain systematically impairs muscle health. Site-specific pain conditions showed variable but substantial effects, with neck/shoulder pain and knee pain demonstrating particularly strong associations (ORs: 0.60–0.73 and 2.88–3.21), indicating that even localized pain can have systemic muscle consequences. Replication analysis supported these findings with 37 significant results (ORs: 0.44–0.97 and 1.32–12.83; Fig. 3B), particularly for MCP and neck/shoulder pain, with minimal heterogeneity and pleiotropy effects observed.

### 3.2. Identification of shared genetic determinants

**Figure 4.**
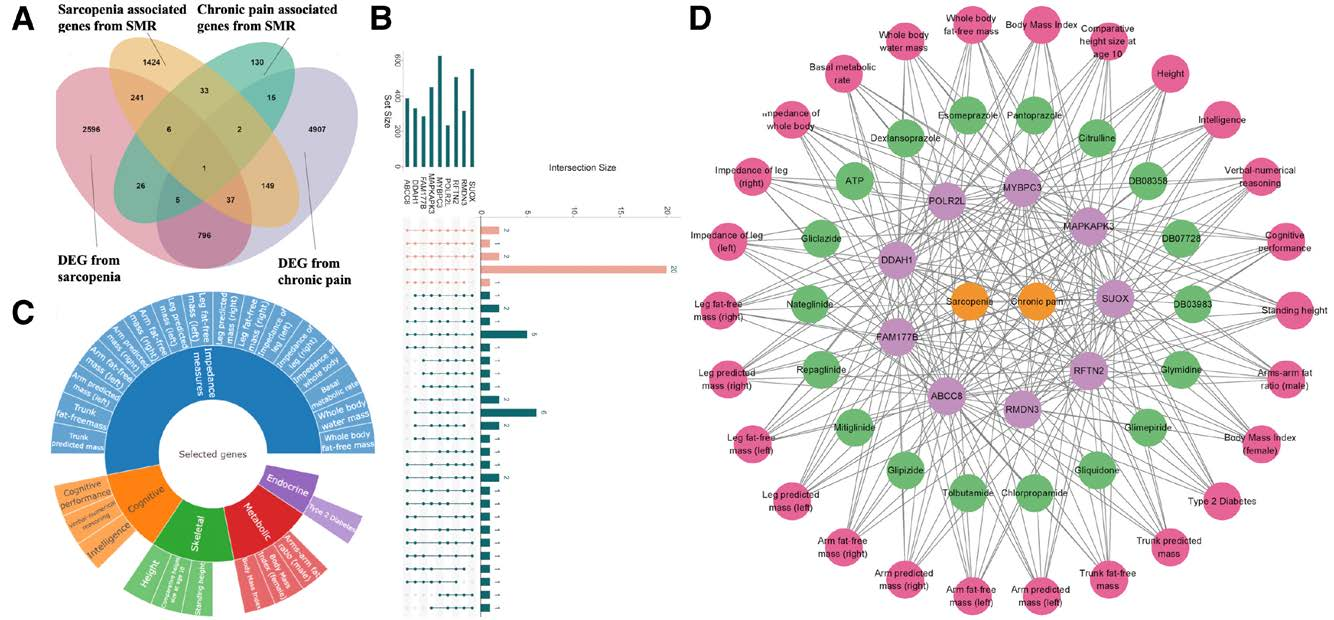
(A) Venn diagram of gene set intersections showing the systematic filtering approach to identify candidate genes from 4 gene sets (summary data-based Mendelian randomization sarcopenia-associated, summary data-based Mendelian randomization chronic pain-associated, differentially expressed gene sarcopenia-related, and differentially expressed gene chronic pain-related), resulting in 51 overlapping candidates and 9 final candidates after colocalization analysis. (B) UpSet plots of phenome-wide association study analysis ordered by degree of overlap, displaying 26 traits associated with at least 8 of the 9 candidate genes across 4756 genome-wide association study datasets encompassing 3302 traits. (C) Sunburst chart of significant traits identified in phenome-wide association study, categorized into 5 groups: impedance measures, cognitive, skeletal, metabolic, and endocrine traits. (D) Interaction network illustrating the complex relationships between diseases, genes, traits, and drugs, highlighting 4 druggable genes (*MAPKAPK3*, *DDAH1*, *ABCC8*, and *SUOX*) associated with 18 compounds, including approved medications and investigational drugs. DEG = differentially expressed gene, SMR = summary data-based Mendelian randomization.

SMR analysis of 3 eQTL datasets (GTEx whole blood, GTEx muscle skeletal, and eQTLgen whole blood) identified 1894 sarcopenia-associated and 218 chronic pain-associated genes (Table S5, Supplemental Digital Content). Colocalization analysis revealed 363 sarcopenia-colocalized and 61 chronic pain-colocalized genes (Table S6, Supplemental Digital Content), with no direct overlap. Differential expression analysis identified 3708 sarcopenia-related and 5912 chronic pain-related DEGs (Table S7 and Fig. S2, Supplemental Digital Content), with 839 genes shared between conditions. We applied a systematic filtering approach to identify candidate genes (Fig. 4A): genes showing overlap across at least 3 of the 4 gene sets (SMR sarcopenia-associated, SMR chronic pain-associated, DEG sarcopenia-related, and DEG chronic pain-related) yielded 51 candidates; subsequent intersection with colocalization results (shown in the Venn diagram section of Fig. 4A) further refined this to 9 final candidates (*MYBPC3*, *POLR2L*, *DDAH1*, *FAM177B*, *ABCC8*, *RMDN3*, *RFTN2*, *SUOX*, and *MAPKAPK3*). Among these, *MAPKAPK3* (mitogen-activated protein kinase-activated protein kinase 3) is particularly notable as it regulates inflammatory responses and stress signaling pathways, providing a mechanistic link between muscle dysfunction and pain perception (Table S8, Supplemental Digital Content).

### 3.3. PheWAS analysis of potential candidate genes

All 9 candidate genes were analyzed using the GWAS Atlas online tool for PheWAS. A total of 4756 GWAS datasets encompassing 3302 traits were analyzed. Our comprehensive analyses revealed that each candidate gene is related to hundreds of traits. The traits associated with the 9 candidate genes exhibited different levels of overlap, with a total of 26 traits associated with at least 8 genes (Fig. 4B). These 26 traits were divided into 5 categories: impedance measures, cognitive, skeletal, metabolic, and endocrine (Fig. 4C). All significant traits are summarized in Table S9, Supplemental Digital Content, serving as references for studying the side effects of potential candidate genes or the association among sarcopenia, chronic pain, and other diseases or phenotypes.

Among the candidate genes, 4 – *MAPKAPK3*, *DDAH1*, *ABCC8*, and *SUOX* – were identified as drug targets associated with 18 compounds. Of these, *DDAH1* is targeted by 4 drugs including 3 approved proton pump inhibitors (pantoprazole, esomeprazole, and dexlansoprazole) and 1 investigational compound (citrulline). *ABCC8* is associated with 11 compounds, primarily consisting of approved antidiabetic medications. *MAPKAPK3* and *SUOX* have been linked to 3 small-molecule compounds in preclinical studies, with *MAPKAPK3* associated with 2 experimental compounds (DB07728 and DB08358) and *SUOX* with 1 (DB03983). Fifteen approved or investigational drugs targeting *DDAH1* and *ABCC8* are detailed in Table 1. The complex relationships between diseases, genes, drugs, and phenotypes are illustrated in Figure 4D.

### 3.4. Pharmacovigilance analysis of AEs associated with candidate drugs

**Figure 5.**
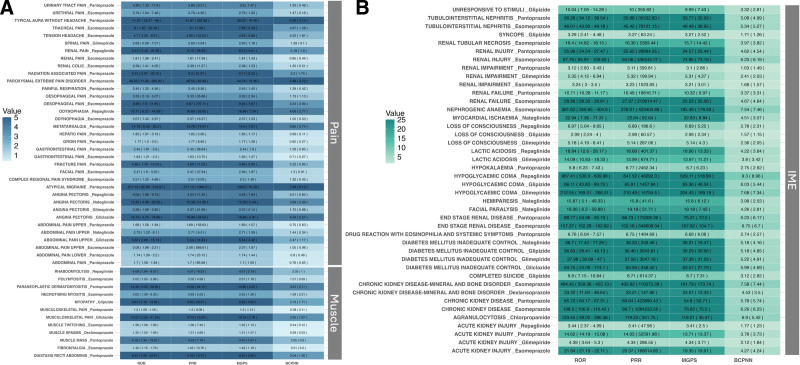
(A) Heatmap of signal values of muscle and pain-related adverse events. (B) Heatmap of signal values of IME adverse events. BCPNN = Bayesian confidence propagation neural network, EBGM = empirical Bayesian geometric mean, PRR = proportional reporting ratio, ROR = reporting odds ratio.

Six drugs – ATP, citrulline, gliquidone, glymidine, mitiglinide, and tolbutamide – were excluded from further analysis due to insufficient sample sizes that prevented computation of reliable signal values. All evaluated drugs were associated with serious AEs; however, muscle-related and pain-related adverse reactions were relatively infrequent (Table S10 and Fig. S3, Supplemental Digital Content). Comparative safety analysis revealed distinct patterns across drug classes (Fig. S4, Supplemental Digital Content). Proton pump inhibitors (esomeprazole, pantoprazole) showed higher frequencies of pain-related and muscle-related AEs, while antidiabetic agents (glimepiride, repaglinide) were associated with serious cardiac and metabolic complications. In contrast, dexlansoprazole and glipizide demonstrated superior safety profiles with minimal muscle-related or pain-related AEs and lower rates of serious complications, supported by adequate sample sizes for reliable assessment. These findings, while limited by the inherent reporting bias and underreporting typical of spontaneous adverse event databases, support dexlansoprazole and glipizide as preferred candidates for further therapeutic investigation (Fig. 5).

### 3.5. Validation of omics data from dexlansoprazole and glipizide

**Figure 6.**
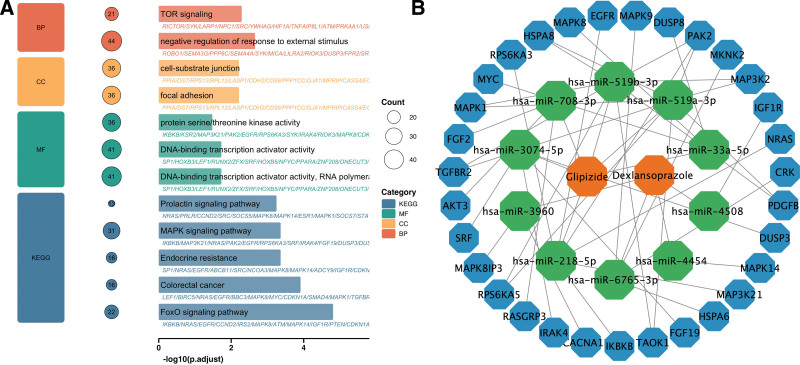
(A) GO and KEGG enrichment analysis of target genes regulated by difference miRNAs. (B) The miRNA regulatory network in the MAPK signaling pathway. BP = biological process, CC = cellular component, GO = Gene Ontology, KEGG = kyoto encyclopedia of genes and genomes, MAPK = mitogen-activated protein kinase, MF = molecular function.

The glipizide treatment samples identified 8 differentially expressed miRNAs corresponding to 988 target genes, while Dexlansoprazole treatment samples showed 5 differentially expressed miRNAs corresponding to 246 target genes (Table S11, Supplemental Digital Content). Figure 6A shows the enrichment results of 1234 target genes GO and kyoto encyclopedia of genes and genomes corresponding to 18 differentially expressed miRNAs, mapped through experimentally validated miRNA-target interactions. The pathway enrichment analysis revealed biologically relevant connections to sarcopenia and chronic pain pathophysiology. The most notable enriched pathways include the mammalian target of rapamycin signaling pathway, mitogen-activated protein kinase (MAPK) signaling pathway and FoxO signaling pathway. These pathways have established clinical relevance: mammalian target of rapamycin signaling is a key therapeutic target in sarcopenia as it regulates muscle protein synthesis and counteracts age-related muscle wasting; MAPK signaling represents a critical intersection mediating both inflammatory muscle catabolism in sarcopenia and central sensitization in chronic pain; and FoxO signaling directly drives muscle protein degradation and atrophy observed in sarcopenic patients. Additionally, we observed enrichment in functions related to focal adhesion and cell-substrate junction, which reflect the muscle structural deterioration characteristic of sarcopenia. Since the MAPK signaling pathway is involved in regulating both muscle and pain simultaneously, its key differential miRNAs and target gene networks are further presented in Figure 6B. These pathway enrichments suggest potential mechanisms through which the selected drugs might influence muscle and pain-related processes, though direct functional validation would be required to confirm therapeutic effects.

## 4. Discussion

Our study provides evidence for a bidirectional causal relationship between sarcopenia and chronic pain through Mendelian randomization analysis of GWAS data from 9 sarcopenia and 22 chronic pain datasets. We identified 9 candidate genes (*DDAH1*, *ABCC8*, *MYBPC3*, *MAPKAPK3*, *ACVR1B*, *ACTN3*, *FTO*, *KLHL30*, and *PIEZO1*) that potentially mediate this relationship, with 4 representing druggable targets. Our genetic analysis identified drug targets associated with both conditions, and pharmacovigilance analysis suggested relatively favorable safety profiles for dexlansoprazole and glipizide among compounds targeting these genes. However, genetic association does not establish clinical efficacy, though experimental validation is required.

Sarcopenia and chronic pain are common conditions in older adults that lead to morbidity and reduced quality of life. A systematic review and meta-analysis found that the overall prevalence of sarcopenia in patients with chronic pain was 11%, with a significantly higher risk of sarcopenia observed in those with chronic pain. The prevalence increased to 21% in individuals with musculoskeletal pain, such as lower back pain.^[34]^ Studies have indicated that patients with chronic pain often experience decreased mobility, leading to the development of sarcopenia.^[35]^ In our study, both bidirectional Mendelian randomization (BMR) and replication BMR found that MCP was associated with all sarcopenia traits, possibly because individuals with more pain sites are more likely to be inactive and with a high risk for falls and fractures, which escalate the development and progression of sarcopenia. Consistently, our results demonstrated that individuals with sarcopenia were more susceptible to chronic pain, especially those with lower hand grip strength, reduced appendicular lean mass, or a slower walking pace. Low muscle strength has gradually gained increasing importance in the diagnosis of sarcopenia instead of the previously emphasized low muscle mass. Consistent with this, in our study, traits related to low muscle strength (HGS [EWGSOP], HGS [FNIH], HGS [right, 2018], HGS [right, 2017], HGS [left, 2018], and HGS [left, 2017]) had a greater positive association rate (59.8%) than ALM (27.3%). In addition, Jin et al found that sarcopenia has a causal relationship with chronic inflammatory conditions such as arthritis.^[36]^ Based on the findings of this study, it can be inferred that patients with sarcopenia might develop chronic inflammation owing to a reduction in muscle mass and function, which in turn could lead to chronic pain. Overall, these findings support the complex bidirectional relationship between sarcopenia and chronic pain.

We integrated GWAS and eQTL data into MR analysis to identify 9 genes that our analysis suggests may be involved in the relationship between sarcopenia and chronic pain. Previous studies have shown that *DDAH1* encodes dimethylarginine dimethylaminohydrolase 1, which degrades asymmetric dimethylarginine, an endogenous inhibitor of nitric oxide synthase (NOS).^[37]^ Literature indicates that *DDAH1* expression increases NOS activity and promotes NO production, and that NO promotes skeletal muscle stem cell division and differentiation via the cGMP pathway^[38,39]^ and influences muscle oxygen and nutrition supply by regulating blood flow. While previous research suggests that chronic pain-induced inflammation can reduce *DDAH1* expression, our findings indicate that *DDAH1* may be involved in the relationship between sarcopenia and chronic pain, though the specific mechanisms require experimental validation. Literature shows that *ABCC8* encodes sulfonylurea receptor 1 (SUR1), an ATP-binding cassette protein responsible for ATP-sensitive K+ channel activity regulation, via which *ABCC8* regulates insulin secretion.^[40,41]^ *MYBPC3* encodes myosin binding protein C (cardiac), a crucial component of cardiac sarcomeres that regulates myocardial contractility and pump function.^[42]^ Our analysis suggests these genes may be involved in the sarcopenia-chronic pain relationship, potentially through metabolic and cardiovascular pathways, though this hypothesis requires further investigation. Our computational analysis suggests potential links between chronic pain and sarcopenia involving metabolic and circulatory pathways. This was further supported by the results of the PheWAS analysis, which not only included traits directly related to muscle quantity and quality but also encompassed several metabolism-related traits, such as type 2 diabetes. Additionally, the results of PheWAS analysis revealed 3 traits related to height, suggesting a potential link between height, sarcopenia, and chronic pain. Consistent with this, previous research has shown that a decrease in height can increase the risk of developing sarcopenia, which in turn leads to chronic pain.^[43]^ The association of these 9 genes with sarcopenia and chronic pain identified in our analysis requires experimental validation to confirm their roles and therapeutic potential.

We identified 4 approved or investigational drugs targeting *DDAH1*: citrulline, pantoprazole, esomeprazole, and dexlansoprazole. Pantoprazole, esomeprazole, and dexlansoprazole are approved drugs that are mainly used for gastrointestinal diseases, such as gastroesophageal reflux disease, erosive esophagitis, and Zollinger–Ellison Syndrome. However, based on real-world adverse event analysis, dexlansoprazole emerged as the optimal potential therapeutic agent, while other drugs such as citrulline require further evaluation due to insufficient data. Notably, citrulline, primarily utilized as a nutritional supplement and classified as a nonessential amino acid, demonstrates potential to enhance muscle function and alleviate fatigue, predominantly through augmentation of nitric oxide production.^[44]^ Beyond its application in ongoing clinical trials for Duchenne and Becker muscular dystrophies, citrulline’s mechanisms may confer benefits in sarcopenia by attenuating muscle loss and inflammatory processes.^[45]^ This collective evidence suggests that dexlansoprazole may warrant further investigation for the management of sarcopenia and chronic pain, though clinical efficacy remains unestablished. Eleven approved or investigational drugs target *ABCC8,* which was mainly developed for type 2 diabetes and associated glycemic control. Tolbutamide, a sulfonylurea drug used for type 2 diabetes, has entered phase I clinical trials for chronic pain treatment by closing ATP-sensitive potassium channels, enhancing insulin secretion, and modulating neural activity.^[46]^ The therapeutic potential of tolbutamide in sarcopenia treatment stems from its capacity to improve metabolic regulation and potentially preserve skeletal muscle mass,^[47]^ while its neural modulatory effects may provide analgesic benefits in chronic pain conditions.^[48]^ For *MAPKAPK3* and *SUOX* targets, 3 compounds are currently undergoing preclinical evaluation. Although limited real-world AEs data exist for glipizide, this second-generation sulfonylurea belongs to the same pharmacological class as tolbutamide but with enhanced potency and improved pharmacokinetic profile. Based on the aforementioned evidence, glipizide showed theoretical associations with both sarcopenia and chronic pain pathways. Despite these encouraging preliminary findings, comprehensive clinical investigations are warranted to establish definitive efficacy and safety profiles for these agents.

This study had several strengths and limitations. The primary strength lies in the comprehensive screening using 9 sarcopenia GWAS datasets and 22 chronic pain GWAS datasets, encompassing multiple phenotypes of these conditions. This extensive dataset contributed to the reliability and completeness of the conclusions drawn. Additionally, the integration of multiple analytical methods, including SMR, Bayesian colocalization, DEG analysis, and pheWAS, corroborated the robustness and reliability of the identified genes. However, this study had notable limitations. Most GWAS data are derived from European populations, significantly limiting generalizability to other ethnic groups. Moreover, our validation analyses utilized temporally expanded datasets with substantial sample overlap rather than truly independent cohorts, which may lead to overestimated effect sizes and inflated statistical significance. Furthermore, while the study successfully identified genes associated with sarcopenia and chronic pain, the lack of experimental or clinical evidence limits our understanding of whether these genetic targets actually modify sarcopenia or pain in humans. The exploratory molecular analysis using GSE216991 was severely limited by single-sample observations (n = 1 each) for candidate drugs in neuronal rather than muscle-relevant tissues, precluding statistical validation and clinical relevance. Although the FAERS database provides real-world data, it suffers from reporting bias, underreporting, and confounding by indication, making therapeutic conclusions highly speculative without clinical validation. The proposed drug targets lack experimental evidence demonstrating their actual efficacy in modifying sarcopenia or pain in humans. Additionally, certain newer pharmaceutical compounds are represented by limited sample sizes. Given the reliance on computational analyses of secondary datasets without independent validation, further rigorous experimental and clinical investigations are essential to validate these findings before any clinical translation can be considered.

## 5. Conclusions

Our study provides evidence for a bidirectional causal relationship between sarcopenia and chronic pain, uncovering a shared genetic architecture that offers insights into their common pathophysiology. By identifying 9 key genes mediating this relationship – with 4 representing druggable targets – we have identified potential avenues that warrant further investigation for therapeutic development. Most significantly, our computational analysis identified 2 FDA-approved compounds (dexlansoprazole and glipizide) as candidates showing pathway-level associations and database safety signals, though these require rigorous experimental validation before clinical consideration. These findings advance our understanding of age-related conditions and suggest potential targets that require prospective clinical trials and experimental validation to confirm therapeutic efficacy before any clinical translation, addressing a critical need in our rapidly aging global population.

## Acknowledgments

The authors thank the IEU Open GWAS project (https://gwas.mrcieu.ac.uk/datasets/), eQTLGen Consortium, FAERS, GEO database (https://www.ncbi.nlm.nih.gov/gds/?term=), and PheWAS for providing open summary results data and online tools for the analyses.

## Author contributions

**Conceptualization:** Jiang Han.

**Data curation:** Juan Li, Jiaqi Huang.

**Formal analysis:** Juan Li, Jiaqi Huang.

**Investigation:** Jiang Han.

**Methodology:** Jiang Han.

**Software:** Juan Li, Jiaqi Huang.

**Supervision:** Jiang Han.

**Validation:** Jiaqi Huang.

**Visualization:** Juan Li, Jiaqi Huang.

**Writing – original draft:** Juan Li, Jiaqi Huang.

**Writing – review & editing:** Jiang Han.





**Figure:**
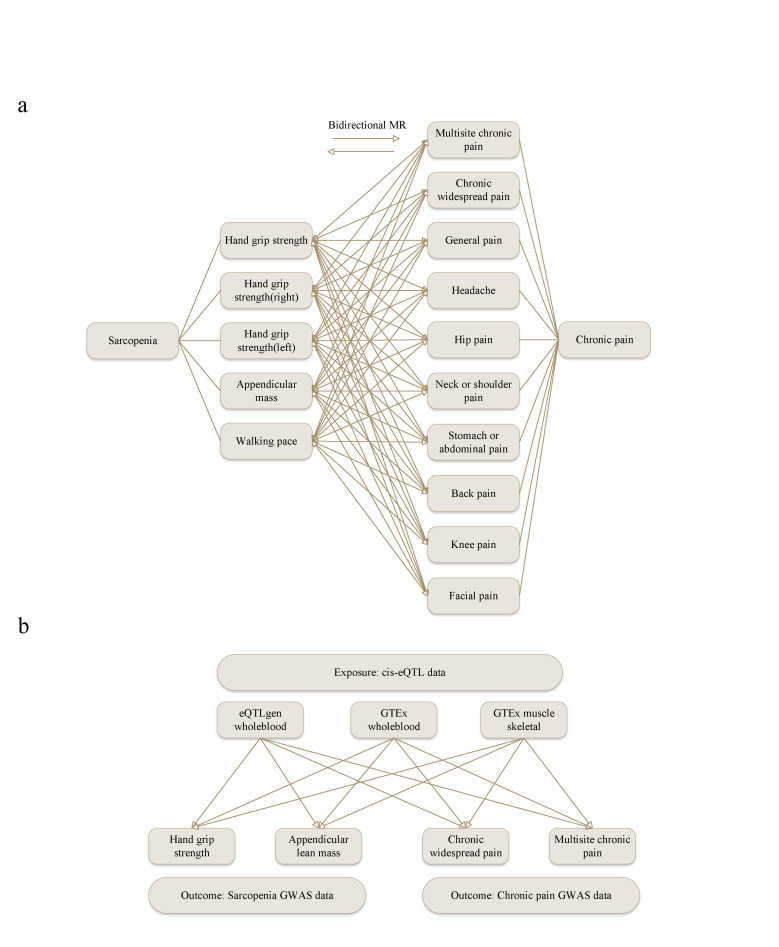












**Figure:**
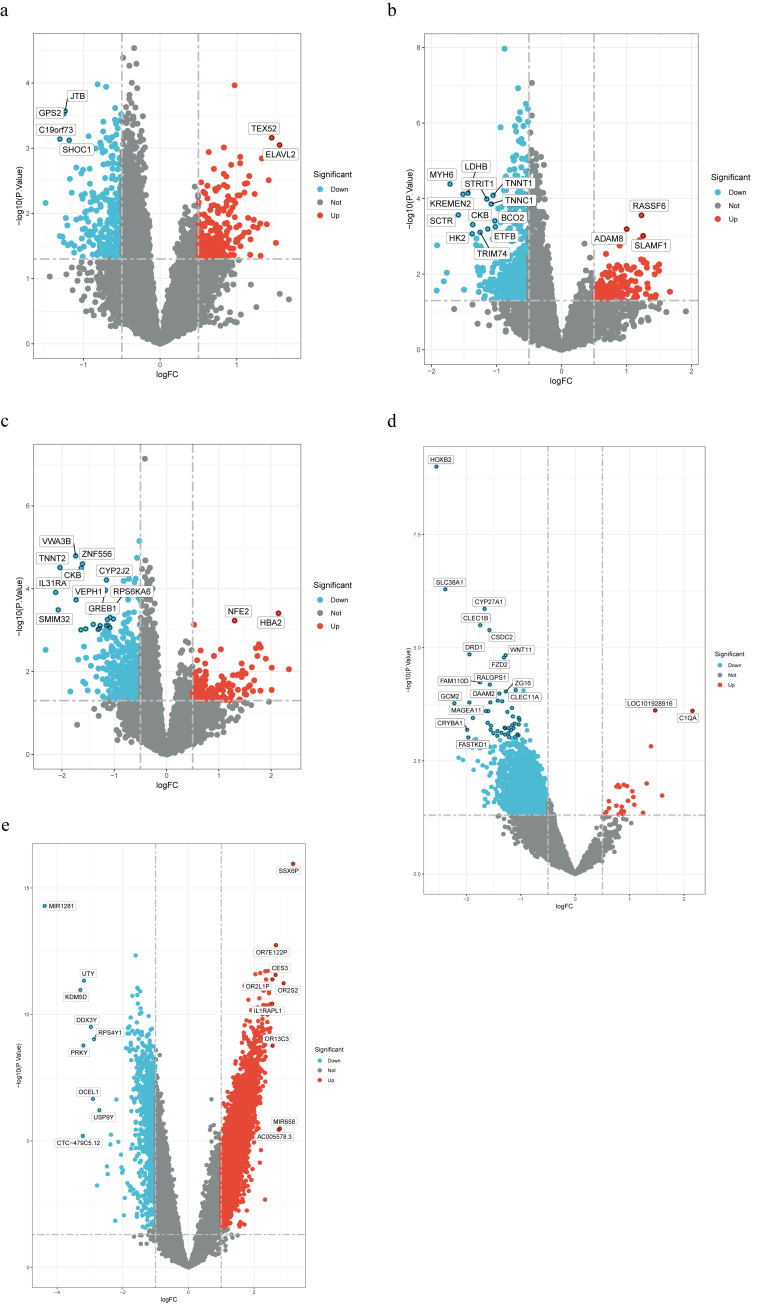








**Figure:**
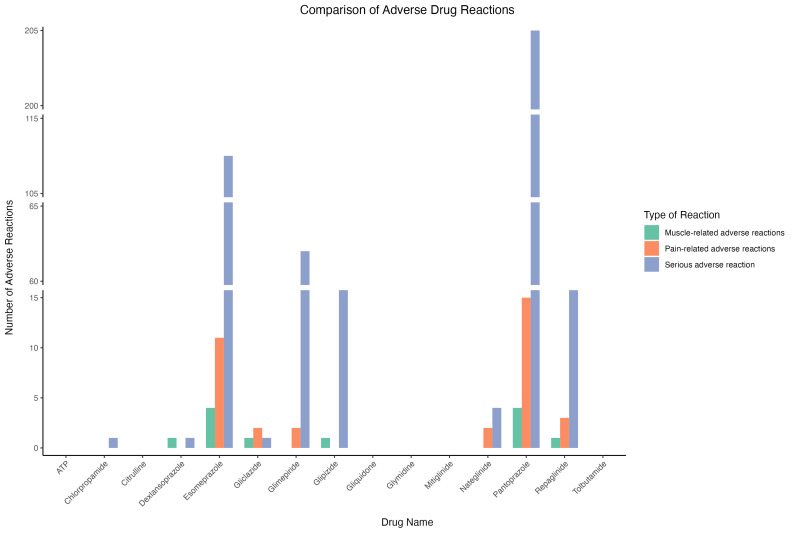


**Figure:**