# Supplementary material for: Sarcopenia and chronic pain: Identification of shared genetic determinants and therapeutic implication
Source: Medicine (Baltimore). 2026 May 15;105(20):e48819. doi: 10.1097/MD.0000000000048819 (PMC13183096; doi:10.1097/MD.0000000000048819)
Supplement: Supplementary file 3 [file medi-105-e48819-s004.docx]

**Table S3: Schematic table of drug analysis and the specific formulas for the four measures.**

| Drugs | Target adverse reaction | Other adverse reaction | Total |
| --- | --- | --- | --- |
| Target drug | a | b | a+b |
| Other drugs | c | d | c+d |
| Total | a+c | b+d | a+b+c+d |

For the above table, acquiring values for variables a, b, c, and d was indispensable. Variable “a” stood for those experiencing desired AEs post target drug, “b” represented individuals with non-target AEs post target drug, “c” denoted individuals experiencing the target AE without target drug, and “d” stood for those with non-target AEs without target drug. The total count N was the sum of a, b, c, and d (N=a+b+c+d).

| Algorithms | Equation | Criteria |
| --- | --- | --- |
| Reporting odds ratio (ROR) | $ROR=(ad)/(bc)$  $SE\left( lnROR \right)=\sqrt{\left( \frac{1}{a}+\frac{1}{b}+\frac{1}{c}+\frac{1}{d} \right)}$  $95\%CI=e^{ln(ROR)\pm1.96\sqrt{\left( \frac{1}{a}+\frac{1}{b}+\frac{1}{c}+\frac{1}{d} \right)}}$ | 95%CI >1, a ≥3 |
| Proportional reporting ratio (PRR) | $PRR=\left[ a(c+d)/c(a+b) \right]$  $SE\left( lnPRR \right)=\sqrt{\left( \frac{1}{a}-\frac{1}{a+b}+\frac{1}{c}-\frac{1}{c+d} \right)}$  $95\%CI=e^{ln(PRR)\pm1.96\sqrt{\left( \frac{1}{a}-\frac{1}{a+b}+\frac{1}{c}-\frac{1}{c+d} \right)}}$ | PRR ≥2, with $\chi^{2}$≥4 and a ≥3 |
| Bayesian confidence propagation neural network (BCPNN) | $IC={log}_{2}\frac{a(a+b+c+d)}{\left( a+b \right)(a+c)}$  $E\left( IC \right)={log}_{2}\frac{(a+\gamma11)(a+b+c+d+\alpha)(a+b+c+d+\beta)}{\left( a+b+c+d+\gamma\right)(a+b+\alpha1)(a+c+\beta1)}$  $V\left( IC \right)=\frac{1}{{(ln2)}^{2}}\left\{ \left[ \frac{\left( a+b+c+d \right)-a+\gamma-\gamma11}{(a+\gamma11)(1+a+b+c+d+\gamma)} \right]+\left[ \frac{\left( a+b+c+d \right)-\left( a+b \right)+\alpha-\alpha1}{(a+b+\alpha1)(1+a+b+c+d+\alpha)} \right]+\left[ \frac{\left( a+b+c+d \right)-\left( a+c \right)+\beta-\beta1}{(a+c+\beta1)(1+a+b+c+d+\beta)} \right] \right\}$  $\gamma=\gamma11\frac{(a+b+c+d+\alpha)(a+b+c+d+\beta)}{\left( a+b+\alpha1 \right)(a+c+\beta1)}$  $IC-2SD=E\left( IC \right)-2\sqrt{V\left( IC \right)}$  *α=β=2, α1=β1=γ11=1* | IC025 (lower limit of 95% CI) >0 |
| Empirical Bayesian Geometric Mean (EBGM) | $EBGM=a(a+b+c+d)/((a+c)(a+b))$  $EBGM05=e^{\ln\left( EBGM \right)-1.64\sqrt{\left( \frac{1}{a}+\frac{1}{b}+\frac{1}{c}+\frac{1}{d} \right)}}$ | EBGM05 (lower limit of 95% CI) >2 |

For the above formulas, acquiring values for variables a, b, c, and d was indispensable. Variable “a” stood for those experiencing desired AEs post target drug, “b” represented individuals with non-target AEs post target drug, “c” denoted individuals experiencing the target AE without target drug, and “d” stood for those with non-target AEs without target drug. The total count N was the sum of a, b, c, and d (N=a+b+c+d).
